# Quality of life in patients with transcatheter aortic valve implantation: an analysis from the INTERVENT project

**DOI:** 10.3389/fcvm.2023.1181771

**Published:** 2023-06-26

**Authors:** Alexander R. Tamm, Marina L. Jobst, Martin Geyer, Omar Hahad, Victoria Buderus, Alexander Schmidt, Jürgen H. Prochaska, Philipp S. Wild, Hendrik Treede, Thomas Münzel, Ralph Stephan von Bardeleben

**Affiliations:** ^1^ Department of Cardiology, Cardiology I, University Medical Center Mainz, Mainz, Germany; ^2^ Department of Cardiology, Preventive Cardiology and Preventive Medicine, University Medical Center Mainz, Mainz, Germany; ^3^ Department of Cardio-Thoracic and Vascular Surgery, University Medical Center Mainz, Mainz, Germany

**Keywords:** aortic stenosis (AS), quality of life, TAVI—transcatheter aortic valve implantation, depression—epidemiology, biobanking

## Abstract

**Background:**

Transcatheter aortic valve implantation (TAVI) is a standard treatment for patients with aortic valve stenosis due to its very low mortality and complication rates. However, survival and physical integrity are not the only important factors. Quality of life (QoL) improvement is a crucial part in the evaluation of therapy success.

**Methods:**

Patients with TAVI were questioned about their QoL before, one month and one year after the intervention as part of the INTERVENT registry trial at Mainz University Medical Center. Three different questionnaires were included in the data collection (Katz ADL, EQ-5D-5l, PHQ-D).

**Results:**

We included 285 TAVI patients in the analysis (mean age 79.8 years, 59.4% male, mean EuroSCORE II 3.8%). 30-day mortality was 3.6%, complications of any kind occurred in 18.9% of the patients. Main finding was a significant increase in the general state of health measured on the visual analog scale by an average of 4.53 (± 23.58) points (BL to 1-month follow-up, *p* = 0.009) and by 5.19 (± 23.64) points (BL to 12-month follow-up, *p* = 0.016). There was also an improvement of depression symptoms, which was reflected in a decrease in the total value of the PHQ-D by 1.67 (± 4.75) points (BL to 12-month follow-up, *p* = 0.001). The evaluation of the EQ-5D-5l showed a significant improvement in mobility after one month (M = −0.41 (± 1.31), *p* < 0.001. Regarding the independence of the patients, no significant difference could be found. Apart from that, patients with risk factors, comorbidities or complications also benefited from the intervention despite their poor starting position.

**Conclusion:**

We could show an early benefit of QoL in TAVI patients with significant improvement in the subjective state of health and a decrease in symptoms of depression. These findings were consistent over 1 year of follow up.

## Introduction

Treatment of severe aortic stenosis has evolved in the past decade with transcatheter aortic valve implantation (TAVI) being the therapy of choice in most patients (1–3). However, the focus is mainly on mortality and complications, while the influence of TAVI on the patients' quality of life (QoL) usually remains unanswered. Especially with younger, healthier patients being treated with TAVI the postinterventional health related QoL is becoming increasingly important (4, 5).

Data on this topic is scarce. Individual studies could show an improvement in terms of QoL with different questionnaire tools and therefore reduced comparability (6–9). In comparison to surgical aortic valve replacement, TAVI patients seem to benefit earlier regarding QoL (10). Mental health and its changes after TAVI are rarely addressed (11).

As part of the INTERVENT project, the aim of this prospective analysis was to compare health related QoL in TAVI patients before and after intervention up to one year of follow-up.

## Methods

### Study design and patient population

Between April 2016 and March 2019, 385 Patients who underwent transfemoral TAVI at the University Medical Center Mainz participated in this study. The data acquisition was part of the „INTERVENT Project“ at the University Medical Center Mainz, a prospective, multicenter, observational cohort study that evaluates interventional procedures for cardiovascular diseases utilizing a large proteomic biobank with the aim to optimize risk stratification and clinical management strategies.

**Figure 1:**
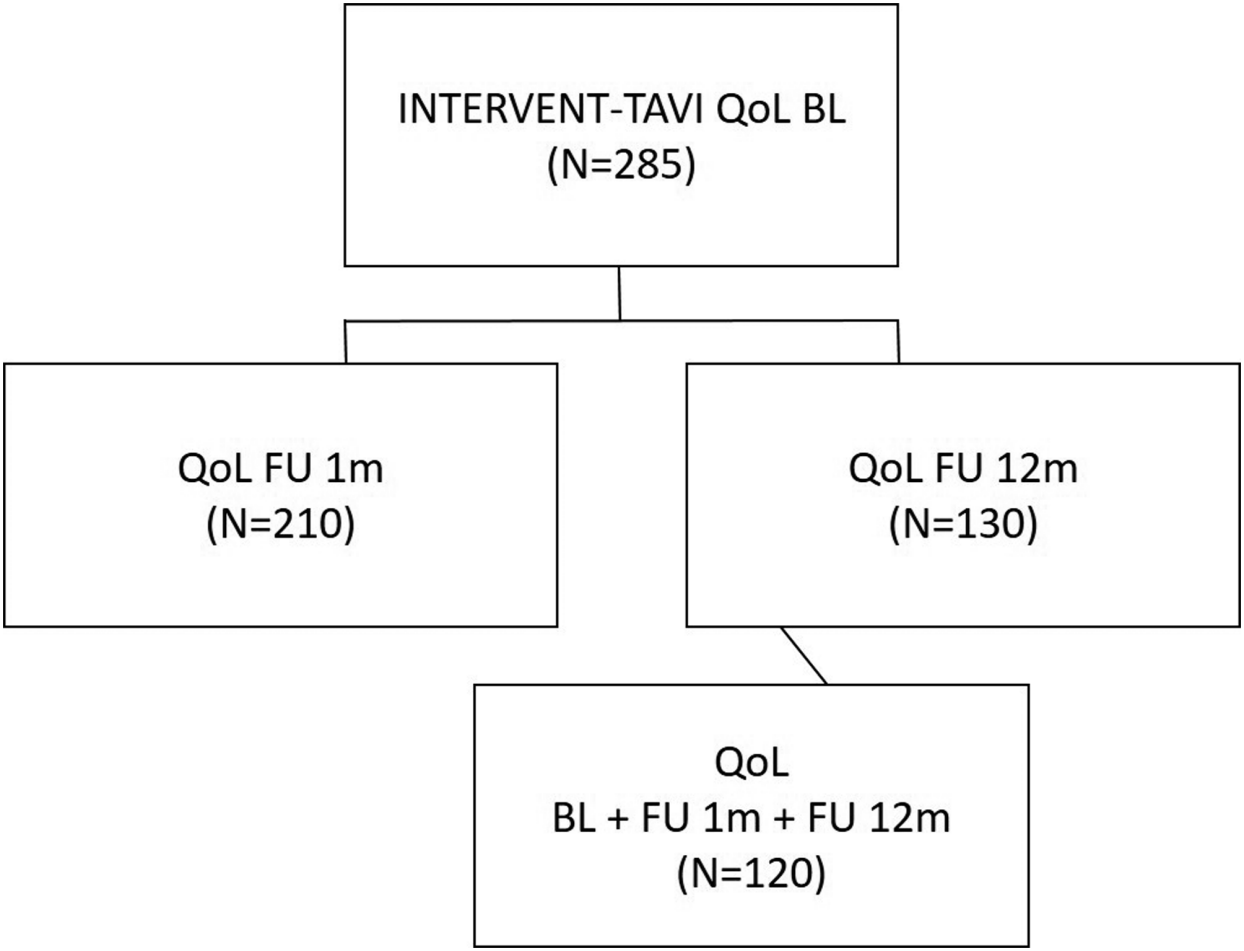
Patient population. A total of 385 patients were included in the INTERVENT project and treated with TAVI at the University Medical Center Mainz from April 2016 until March 2019. 285 patients that completed the baseline QoL Questionnaires could be included in the analysis. TAVI, transcatheter aortic valve implantation; QoL, quality of life; BL, baseline; FU1M, follow-up after 1 month; FU12M, follow-up after 12 months; QoL, quality of life.

A total of 285 patients were interviewed about their quality of life before TAVI, at 1 month (*n* = 210) and 12 months (*n* = 130) after undergoing the intervention (Figure 1). Three questionnaires were used to provide a comprehensive description of the quality of life (see below). The patients participated on a voluntary basis. Before the start of the study approval was granted by the local ethics committee [reference number 837.224.13 (8909-F)]. The following factors were required for participation in the study: severe aortic valve stenosis (AVA ≤ 1 cm^2^ or *Δ*p ≥ 40 mmHg), presence of symptoms (NYHA class III/IV or angina pectoris) and anatomical suitability for TAVI. Outcome data was assessed regarding VARC-3 criteria (12). The patients were followed up at 30 days (FU1M) and 1 year (FU12M).

### QoL questionnaires

For a comprehensive picture of the patients' health related quality of life, three different questionnaires were included.

The Katz Index of Independence in Activities of Daily Living (Katz ADL) served as a measure of self-reliance (13). This standardized questionnaire has been used worldwide for decades and contains 6 items to determine the patient's (in)dependence. Despite not being a „classic” QoL questionnaire, it is an important tool for predicting the potential additional burden on the patient and the healthcare system due to an increased need for care.

General health was determined using the European Health Questionnaire EQ-5D-5l and the visual analogue scale. This questionnaire, published by the EuroQol Research Foundation, is characterized by high reliability and validity (14, 15). Its 5 dimensions reflect both physical and mental impairments of the patient. Due to the lack of leading questions, the visual analog scale proved to be a particularly individual measuring tool of the subjectively perceived state of health.

The mental health of the patients was analysed using the short version of the Patient Health Questionnaire in German (PHQ-D) by Löwe et al. (16, 17). Employing the associated manual, the presence of a major depressive or other depressive syndrome could be detected in addition to a general assessment of the mental status. Furthermore, a panic syndrome and psychosocial functioning could be examined.

### Statistics

Statistical analysis was performed using SPSS software (IBM® SPSS® statistics, version 26 for Mac). Continuous variables were expressed as mean ± SD when normally distributed, otherwise as median and interquartile ranges. Categorical variables were presented as frequencies and percentage, unless otherwise specified. Shapiro–Wilk test was used to assess normality for continuous data. Statistical significance was assessed using a t-test in normally distributed data or a Mann-Whitney-U test in non-normally distributed data. Chi-square test was used to compare categorical variables. All statistical tests were two-sided and *p* < 0.05 was considered to be statistically significant.

## Results

### Baseline characteristics

The total cohort included 285 patients (59.4% male) with a mean patient age of 79.8 ± 5.6 years and a mean EuroSCORE II of 3.8 ± 3.7%. 58.6% of the patients had coronary artery disease (CAD) as a preexisting condition, 39.1% had diabetes mellitus and 24.7% had atrial fibrillation. 15.9% had a history of myocardial infarction, 13.4% had a previous stroke/TIA. In this patient collective, the mean AVA was 0.8 ± 0.2 cm^2^, the P_mean_ was 36.8 ± 14.4 mmHg and the LVEF averaged 56.0 ± 11.4% (Table 1).

**Table 1:** Patient characteristics.

Baseline Characteristics		*n*
Age (years)	79.8 ± 5.6	285
Male sex—*n* (%)	168 (59.4)	285
BMI (kg/m2)	27.7 ± 5.1	274
EuroSCORE II (%)	3.8 ± 3.7	182
NYHA Class III or IV—*n* (%)	97 (64.7)	150
Coronary artery disease—*n* (%)	163 (58.6)	278
Previous myocardial infarction—*n* (%)	44 (15.9)	276
Previous stroke—*n* (%)	37 (13.4)	277
Cerebrovascular disease—*n* (%)	52 (33.3)	156
Peripheral artery disease—*n* (%)	36 (14.4)	250
COPD—*n* (%)	78 (28.2)	277
Chronic Kidney Injury—*n* (%)	64 (23.2)	276
Diabetes—*n* (%)	108 (39.1)	276
Current smoker—*n* (%)	19 (6.9)	276
Atrial fibrillation—*n* (%)	64 (24.7)	259
Permanent pacemaker—*n* (%)	30 (10.8)	278
Frailty—*n* (%)	37 (17.4)	213
AVA—*n* (%)	0.8 ± 0.2	193
Peak pressure gradient (mmHg)	62.3 ± 22.6	192
Mean pressure gradient (mmHg)	36.8 ± 14.4	193
LVEF—%	56.0 ± 11.4	190

BMI, Body-Mass-Index; NYHA, New York Heart Association; COPD, chronic obstructive pulmonary disease; AVA, aortic valve area; LVEF, left ventricular ejection fraction.

Regarding the level of independence, the patients achieved a mean value of 5.7 ± 0.9 out of a maximum of 6.0 points in the Katz ADL before the intervention. The EQ-5D-5l was evaluated according to the official Index Value Calculator of the EuroQol Research Foundation for the German population and averaged 0.8 ± 0.2 (with a range of −0.205 to 1.0). The preinterventional EQ-VAS averaged 61.1 ± 21.1 (with a range of 0–100). Concerning mental health, the cohort showed a total value of the PHQ-D of 6.2 ± 4.8, which corresponds to a mild depressive disorder according to the user manual. Pursuant to the evaluation by Löwe et al, 10.1% of the patients had a major depressive syndrome and 10.1% had a minor depressive syndrome. A panic syndrome was found in 1.2% of the patients (Table 2).

**Table 2:** Health related quality of life at baseline.

		*n*
Katz ADL Index—total points (0–6)	5.7 ± .9	278
EQ-5D-5l—U value (−0.661–1.0)^a^	.8 ± .3	282
EQ-5D-5l—V value (−0.205–1.0)^b^	.8 ± .2	282
EQ-VAS—visual analog scale (0–100)	61.1 ± 21.1	275
PHQ-D: total points (0–27)	6.2 ± 4.8	248
PHQ-D: depressive symptoms—*n* (%)^c^	54 (21.8)	248
PHQ-D: major depressive syndrome—*n* (%)	25 (10.1)	248
PHQ-D: mild depressive syndrome—*n* (%)	25 (10.1)	248
PHQ-D: panic syndrome—*n* (%)	3 (1.2)	246
PHQ-D: limitation of psycho-social function^d^—*n* (%)	52 (21.9)	237

^a^ based on Ludwig et al. (2018).

^b^ based on official Index Value Calculator of EuroQol Research Found.

^c^ PHQ sum value ≥10.

^d^ moderate or severe impairment in every day social interaction, housekeeping, or work.

ADL, activities of daily living; PHQ-D, patient health questionnaire (short version); VAS, visual analog scale.

### Outcome

The intervention was technically successful in 99.5%. The majority of patients (*n* = 196, 88,3%) were treated in general anesthesia. Periinterventional complications of any kind occurred in 18.9%, of which vascular complications of any kind were predominating (12.5%). Permanent pacemaker implantation was needed in 42 patients (19.0%). One patient (0.4%) died during the hospital stay. Three patients (1.7%) suffered from a stroke after the intervention. At one-month FU, the rehospitalization rate was 17.9% and the mortality rate was 3.6%. At 12 months, 40.2% had been hospitalized again, 7.2% had died (Table 3).

**Table 3:** Technical success, complications, rehospitalisation and death.

		*n*
Technical success of the intervention—number (%)	220 (99.5)	221
Periinterventional complications—number (%)	42 (18.9)	222
Death during hospitalisation—number (%)	1 (.4)	257
Permanent Pacemaker Implantation post intervention (%)	42 (19.0)	221
Vascular complications (all)—number (%)	22 (12.5)	176
New onset AF—number (%)	11 (9.5)	116
Stroke—number (%)	3 (1.7)	176
Residual aortic regurgitation—number (%)	3 (1.4)	219
Rehospitalization until FU1M—number (%)	44 (17.9)	246
Death until FU1M—number (%)	9 (3.6)	247
Rehospitalization until FU12M—number (%)	84 (40.2)	209
Death until FU12M—number (%)	15 (7.2)	209

AVB, atrioventricular block; FU1M, follow-up after 1 month; FU12M, follow-up after 12 months; LBBB, left bundle branch block; AF, atrial fibrillation.

### Quality of life

Regarding the very high level of preinterventional independence, there was hardly any change postinterventional: the average Katz ADL was 5.6 ± 1.0 at 30 days and 5.7 ± 0.8 one year after the intervention (Table 4).

**Table 4:** QoL—mean values over time.

	BL	FU1M	FU12M
Katz ADL Index—total score (0–6)	5.7 ± 0.9	5.6 ± 1.0	5.7 ± 0.8
	(*n* = 278)	(*n* = 194)	(*n* = 126)
EQ-5D-5l—U-value^a^ (−0.661–1.0)	.8 ± .3	.8 ± .3	.8 ± .3
	(*n* = 282)	(*n* = 201)	(*n* = 128)
EQ-5D-5l—V-value^b^ (−0.205–1.0)	.8 ± .2	.8 ± .2	.8 ± .2
	(*n* = 282)	(*n* = 201)	(*n* = 128)
EQ-VAS—number (0–100)	61.1 ± 21.1	66.4 ± 20.7	66.1 ± 19.9
	(*n* = 275)	(*n* = 194)	(*n* = 127)
PHQ-D: total value (0–27)	6.2 ± 4.8	3.5 ± 4.4	4.8 ± 4.3
	(*n* = 248)	(*n* = 43)	(*n* = 118)
PHQ-D: major depressive syndrome^c^—%	10.1	7.0	5.9
	(*n* = 248)	(*n* = 43)	(*n* = 118)
PHQ-D: other depressive syndrome^c^—%	10.1	0.0	5.1
	(*n* = 248)	(*n* = 43)	(*n* = 118)
PHQ-D: panic syndrome^c^—%	1.2	0.0	1.8
	(*n* = 246)	(*n* = 42)	(*n* = 112)

^a^ Weighting according to Ludwig et al. (2018).

^b^ Weighting according to the official Index Value Calculator of the EuroQol Research Foundation.

^c^ According to the manual by Ludwig et al. (2002).

ADL, Activities of Daily Living; BL, Baseline; FU1M, Follow-up after 1 month; FU12M, Follow-up after 12 months; PHQ-D, Patient Health Questionnaire (short form); QoL, Quality of Life; VAS, Visual Analogue Scale.

Similarly, the general health status in its weighted variant (see above) showed no change. However, the differentiated analysis of the 5 dimensions revealed that the mobility of the patients improved significantly within the first month after the intervention [BL vs. FU1M M = −0.41 (±1.31), p < 0.001], while there was a slight but significant deterioration in pain or physical complaints within the first year after the intervention [BL vs. FU12M M = 0.20 (±1.10), *p* = 0.044].

**Figure 2:**
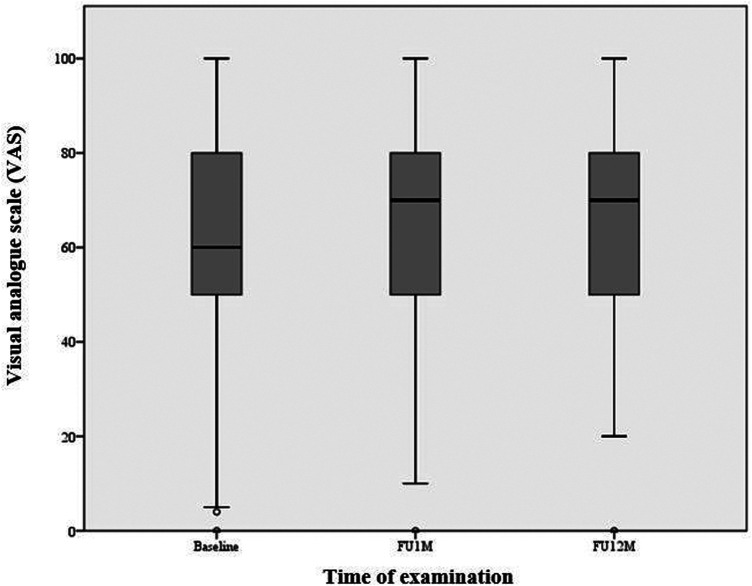
Subjective state of health (EQ-VAS). A significant improvement can be observed in the subjective assessment of the general state of health using the visual analogue scale (VAS). Within the first month after the intervention, there was a significant increase by an average of 4.53 ± 23.58 points [95% CI (1.16, 7.89), *p* = 0.009]. After one year, the value improved by 5.19 ± 23.64 points compared to the baseline examination [95% CI (0.97, 9.41), *p* = 0.016]. Box plots indicate minimum, maximum, 25th percentile, median, and 75th percentile.

A significant improvement was shown in the subjective assessment of the general state of health using the visual analogue scale. Within the first month after the intervention, there was a significant increase by an average of 4.53 ± 23.58 points [95% CI (1.16, 7.89), *p* = 0.009]. After one year, the value improved by 5.19 ± 23.64 points compared to the baseline examination [95% CI (0.97, 9.41), *p* = 0.016] (Table 5, Figure 2).

**Table 5:** QoL change at follow-Up—paired analysis.

		M (±SD)	T	df	*p*
1	Katz ADL Index	−.10 (±1.05)	−1.30	190	.194
	Baseline—FU1M				
2	Katz ADL Index	−.74 (±.73)	−1.12	120	.266
	Baseline—FU12M				
3	Katz ADL Index	.08 (±.71)	1.22	107	.227
	FU1M—FU12M				
4	EQ-5D-5l—U-value^a^	.03 (±.26)	1.42	198	.158
	Baseline—FU1M				
5	EQ-5D-5l—U-value^a^	−.01 (±.28)	−.42	124	.679
	Baseline—FU12M				
6	EQ-5D-5l—U-value^a^	−.01 (±.18)	−.44	113	.659
	FU1M– FU12M				
7	EQ-5D-5l—V-value^b^	.03 (±.21)	1.99	198	.048*
	Baseline—FU1M				
8	EQ-5D-5l—V-value^b^	−.01 (±.22)	−.66	124	.508
	Baseline—FU12M				
9	EQ-5D-5l—V-value^b^	−.01 (±.15)	−.88	113	.382
	FU1M—FU12M				
10	EQ-VAS	4.53 (±23.58)	2.65	190	.009*
	Baseline—FU1M				
11	EQ-VAS	5.19 (±23.64)	2.43	122	.016*
	Baseline—FU12M				
12	EQ-VAS	.61 (±19.48)	.32	106	.748
	FU1M—FU12M				
13	PHQ-D total value	−1.21 (±5.17)	−1.52	41	.136
	Baseline—FU1M				
14	PHQ-D total value	−1.67 (±4.75)	−3.57	102	.001*
	Baseline—FU12M				
15	PHQ-D total value	−.375 (±1.77)	−.060	7	.567
	FU1M—FU12M				
16	PHQ-D psychosocial funct. capacity	−.25 (±.84)	−1.88	39	.067
	Baseline—FU1M				
17	PHQ-D psychosocial funct. capacity	−.28 (±1.14)	− 2.24	80	.028*
	Baseline—FU12M				
18	PHQ-D psychosocial funct. capacity	.13 (±.64)	.55	7	.598
	FU1M—FU12M				

* significant (*p* < 0.05).

^a^ Weighting according to Ludwig et al. (2018).

^b^ Weighting according to the official Index Value Calculator of the EuroQol Research Foundation.

ADL, Activities of Daily Living; BL, Baseline; FU1M, Follow-up after 1 month; FU12M, Follow-up after 12 months; PHQ-D, Patient Health Questionnaire (short form); QoL, Quality of Life; VAS, Visual Analogue Scale.

**Figure 3:**
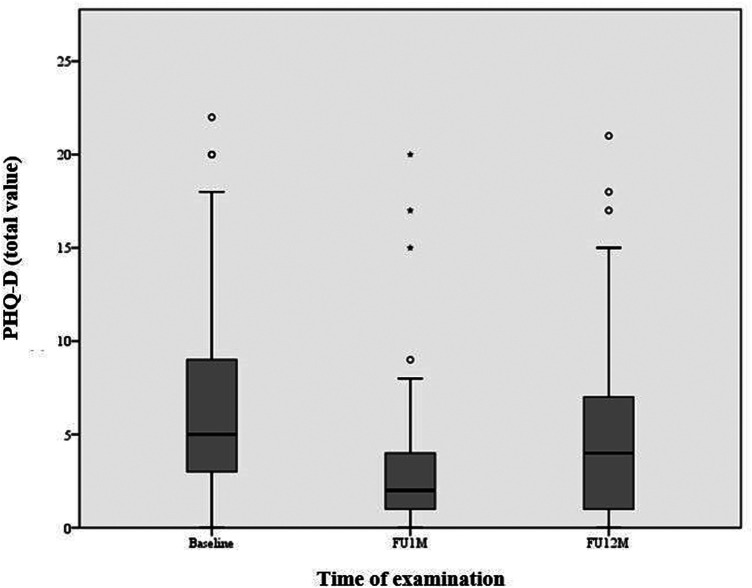
Depression symptoms (PHQ-D). A significant improvement in symptoms of depression, reflected in a 1.67 ± 4.75 point decrease in PHQ-D total score after one year compared to baseline [95% CI (−2.60, −0.74), *p* = 0.001]. Box plots indicate minimum, maximum, 25th percentile, median, and 75th percentile.

**Figure 4:**
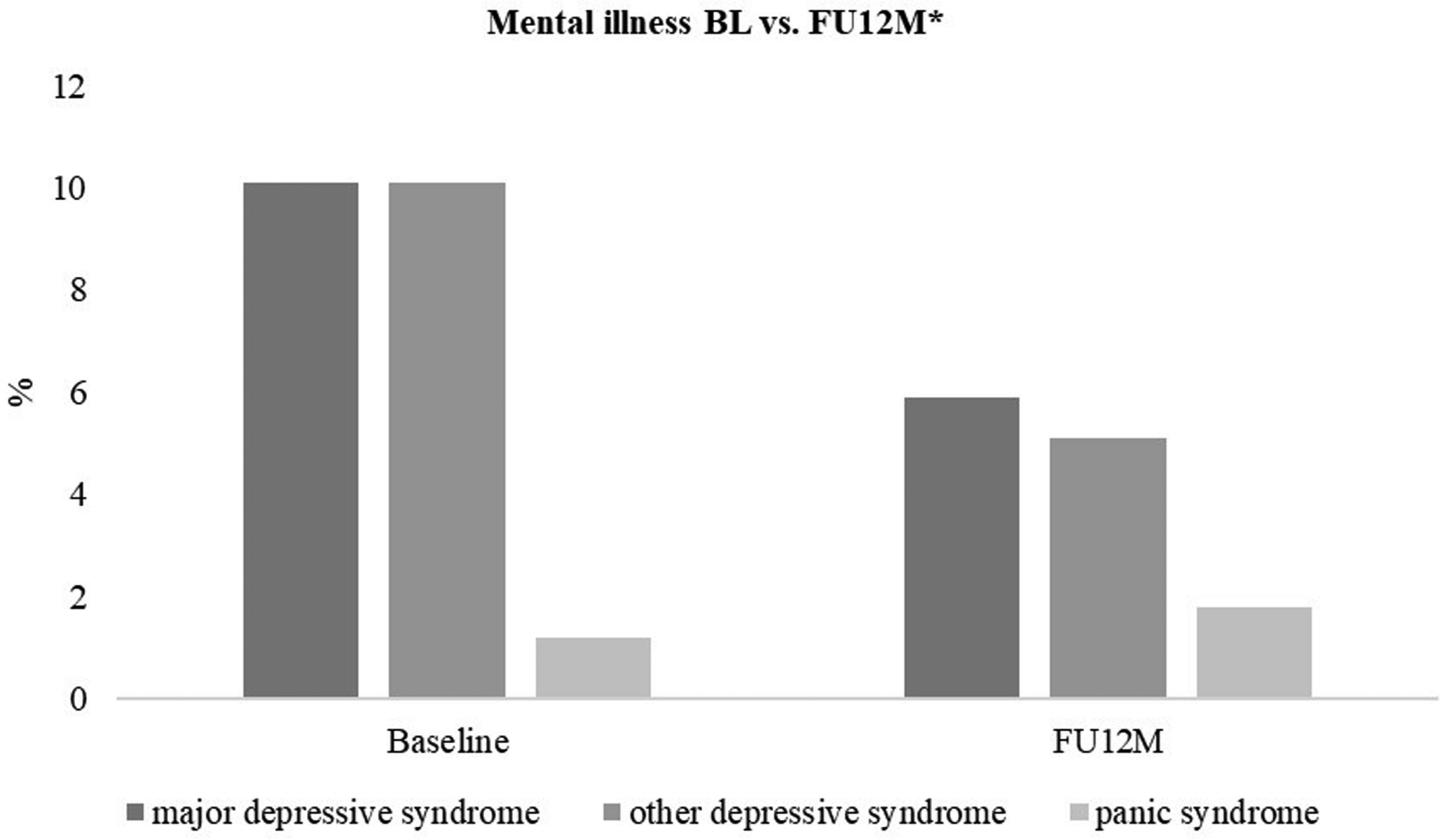
Mental illness (PHQ-D). There was a decrease in major depressive syndrome from 10.1% (*n* = 248) to 5.9% (*n* = 118) and in other depressive syndrome from 10.1% (*n* = 248) to 5.1% (*n* = 118). An increase in panic syndrome from 1.2% (*n* = 246) to 1.8% (*n* = 112) was observed. * according to the PHQ-D manual by Löwe et al. (2002).

There was also a significant improvement in symptoms of depression, reflected in a 1.67 ± 4.75 point decrease in PHQ-D total score after one year compared to baseline [95% CI (−2.60, −0.74), *p* = 0.001]. In addition, there was a decrease in major depressive syndrome from 10.1% (*n* = 248) to 5.9% (*n* = 118) and in mild depressive syndrome from 10.1% (*n* = 248) to 5.1% (*n* = 118) (Tables 4, 5; Figures 3, 4).

Subgroup analysis showed worse baseline QoL in patients that were female (PHQ-D 7.0 ± 4.7 vs. 5.7 ± 4.7, *p* = 0.040), over 80 years old (EQ-VAS 58.2 ± 22.1 vs. 63.7 ± 20.0, *p* = 0.031; PHQ-D 6.9 ± 5.2 vs. 5.5 ± 4.2, *p* = 0.021) or with higher surgical risk estimated by EuroSCORE II over 4.0% (EQ-VAS 49.1 ± 22.4 vs. 63.0 ± 20.8, *p* < 0.001; PHQ-D 7.6 ± 4.7 vs. 5.6 ± 4.4, *p* = 0.024)(Supplementary Table S1). All patients benefited in terms of depression symptoms over 1 year, while especially younger, male and low risk patients showed an improvement in the subjective QoL measured by EQ-VAS (Supplementary Table S2).

## Discussion

In this study we aimed to evaluate changes in patient's health related QoL in a real-world TAVI cohort. Our analysis showed a significant improvement in the subjective state of health and a decrease in symptoms of depression already at short term and consistent over 1 year of follow-up. Subgroups that benefited most were young, male, and low-risk patients. Although rehospitalization rate was rather high with 40.2% at 1 year, this seemed not to affect the improvement in QoL. A possible explanation would be the heterogeneity of admission reasons which included also planned examinations.

We included patients with a mean age of 79.8 ± 5.6 years and a mean EuroSCORE II of 3.8 ± 3.7%, representing an elderly, low-to intermediate risk, real-world cohort. Compared to the PARTNER-3 and Evolute Low Risk Study, our pre-interventional collective presented with slightly worse conditions (2, 3). This was reflected in a higher average age, greater general risk and lower LVEF. After the intervention, the rates of rehospitalization, complications and mortality were comparatively higher in our study. It is conceivable that the poorer starting conditions contributed in part to this difference, however, further investigations in this regard would be interesting.

The baseline values of the Katz ADL and the weighted EQ-5D-5l were very high, leaving little room for improvement. Over the course of the year, these values remained largely unchanged, which can be seen as positive in this context. A differentiated analysis of the EQ-5D-5l showed a significant increase in mobility after one month. A study of the German Aortic Valve Registry (GARY) from 2016 delivered a comparable result (8). It can therefore be assumed that this improvement is directly related to the intervention. However, there was a slight but significant deterioration in pain or discomfort after one year, while the GARY scores remained constant. Since pain is a common problem in older age (up to 80%), the direct connection between the intervention and the increase is questionable (18).

A significant increase in subjective health, measured using the VAS, was already evident at the one-month follow-up (see above), so that a direct connection between the intervention and the improvement can be assumed. Even after one year, this value was mostly constant (difference between FU1M and FU12M: 0.61 points). This positive development coincides with the results of other studies, e.g., the GARY (52.6 to 59.6) and a study by the University of Jena (46.7 to 55.3) (8, 19). It is interesting that in women, older (> 80 years) and high-risk patients (EuroSCORE II > 4%) compared to the other collective, the improvement was of a smaller extent and did not reach the level of significance. The reason for this could be a smaller sample size in the named collectives. Furthermore, older and high-risk patients showed significantly lower values in the VAS even before the intervention. It is therefore reasonable to assume that poorer baseline values could lead to a smaller improvement. Apart from this, the fact that the younger patients showed a significant and greater improvement in the VAS compared to the older ones is of particular interest since for most low-risk patients in Germany under 75 years of age, the SAVR is still favored.

Regarding the sex-related difference, we should keep in mind that a direct comparison of men and women about HRQoL is not appropriate. In addition to various biological differences, the main reason for this is differences in the perception and presentation of the own state of health (20, 21). The tendency towards improvement can thus be interpreted as positive, but the differences between men and women cannot be evaluated without gender-specific questions and elimination of all confounders.

This fact must also be considered when interpreting the results of the depression questionnaire. Here, too, the male patients showed significantly better values before the intervention and a significant improvement just one month later, while the female patients showed a non-significant deterioration during this period. Apart from the fact that female gender is considered a general risk factor for the development of depression, the already mentioned different perception and expression of the emotions also influence the results here (20–25). In addition, at the annual follow-up, a significant improvement was recorded in both collectives. Thus, it cannot be assumed that women do not benefit or benefit to a lesser extent from the intervention in terms of their mental state. Overall, despite the below-average condition before the intervention (PHQ-D on average 6.2 out of 27 points, corresponding to mild depressive symptoms), the patient collective showed a significant improvement and consecutive decrease in the prevalence of depressive syndromes after one year. This result is comparable to other studies and suggests that the improved quality of life after TAVI has a real impact on patients’ mental health (11, 19, 26).

## Limitations

This study reflects a “real world” patient population enrolled in an observational study without independent adjudication, therefore typical limitations apply. Due to the single-arm design with no conservative or surgical control group, no statement on comparability to these populations can be made. The analyzed cohort size of 285 patients is inferior to large registry studies or randomized trials, so results cannot be translated to the general population of AS patients.

Since QoL questionnaires were handed out on a voluntarily base, the number of evaluable data was reduced over follow-up time, and thus might bias the results. Also, patients often did not complete all possible questionnaires, which could further affect the analysis.

Furthermore, a QoL questionnaire has the inherent bias of the patient's subjective view. Although analyzed by standard definitions, this lack of objectivity is a limitation regarding data collection and interpretation.

## Conclusions

We could show an early benefit of QoL in TAVI patients with significant improvement in the subjective state of health and a decrease in symptoms of depression. These findings were consistent over 1 year of follow up and were independent of patients' gender, age or condition before the intervention. Our findings, especially regarding mental health have to be reproduced in further studies.

## Data Availability

The raw data supporting the conclusions of this article will be made available by the authors, without undue reservation.
