# Supplementary material for: Quality of life in patients with transcatheter aortic valve implantation: an analysis from the INTERVENT project
Source: Front Cardiovasc Med. 2023 Jun 26;10:1181771. doi: 10.3389/fcvm.2023.1181771 (PMC10330696; doi:10.3389/fcvm.2023.1181771)
Supplement: Supplementary file 1 [file Table1.docx]

**Supplement Table 1**  **Baseline QoL by age, sex and risk**

|  | **EQ-VAS** | **PHQ-D** |
| --- | --- | --- |
| Age ≤ 80 y. | 63.7 ± 20.0  (n = 144) | 5.5 ± 4.2  (n = 134) |
| Age > 80 y. | 58.2 ± 22.1  (n = 129) | 6.9 ± 5.2  (n = 112) |
| mean difference | 5.53 | -1.40 |
| p | **.031** | **.021** |
| Male | 62.0 ± 21.4  (n = 165) | 5.7 ± 4.7  (n = 152) |
| Female | 59.7 ± 20.8  (n = 108) | 7.0 ± 4.7  (n = 94) |
| mean difference | 2.34 | -1.28 |
| p | .373 | **.040** |
| EuroSCORE II < 4% | 63.0 ± 20.8  (n = 136) | 5.6 ± 4.4  (n = 126) |
| EuroSCORE II ≥ 4% | 49.1 ± 22.4  (n = 40) | 7.6 ± 4.7  (n = 31) |
| mean difference | 13.89 | -2.04 |
| p | **.000** | **.024** |

PHQ-D = patient health questionnaire (short version), VAS = visual analog scale

**Supplement Table 2**  **QoL Baseline vs. FU12M by age, sex and risk**

|  | **EQ-VAS**  **BL – FU12M** | **PHQ-D**  **BL – FU12M** |
| --- | --- | --- |
| Age ≤ 80 y. | 7.74 ± 22.05  (n = 73) | -1.38 ± 4.59  (n = 64) |
| p | **.004** | **.020** |
| Age > 80 y. | 1.46 ± 25.56  (n = 50) | -2.15 ± 5.01  (n = 39) |
| p | .688 | **.011** |
| Male | 6.89 ± 24.64  (n = 74) | -1.65 ± 4.99  (n = 63) |
| p | **.019** | **.011** |
| Female | 2.61 ± 22.03  (n = 49) | -1.70 ± 4.40  (n = 40) |
| p | .411 | **.019** |
| EuroSCORE II < 4% | 5.44 ± 21.78  (n = 86) | -1.07 ± 4.32  (n = 73) |
| p | **.023** | **.038** |
| EuroSCORE II ≥ 4% | 9.13 ± 26.70  (n = 23) | -3.82 ± 6.47  (n = 17) |
| p | .115 | **.027** |

BL = Baseline, FU12M = follow-up after 12 months, PHQ-D = patient health questionnaire (short version), VAS = visual analog scale
